# Superconducting gap anisotropy sensitive to nematic domains in FeSe

**DOI:** 10.1038/s41467-017-02739-y

**Published:** 2018-01-18

**Authors:** Takahiro Hashimoto, Yuichi Ota, Haruyoshi Q. Yamamoto, Yuya Suzuki, Takahiro Shimojima, Shuntaro Watanabe, Chuangtian Chen, Shigeru Kasahara, Yuji Matsuda, Takasada Shibauchi, Kozo Okazaki, Shik Shin

**Affiliations:** 1 Institute for Solid State Physics (ISSP), University of Tokyo, Kashiwa, Chiba 277-8581 Japan; 2 Quantum-Phase Electronics Center (QPEC) and Department of Applied Physics, University of Tokyo, Bunkyo, Tokyo 113-8656 Japan; 3 Research Institute for Science and Technology, Tokyo University of Science, Chiba, 278-8510 Japan; 4 Beijing Center for Crystal R&D, Chinese Academy of Science (CAS), Zhongguancun, Beijing, 100190 China; 5 Department of Physics, Kyoto University, Kyoto, 606-8502 Japan; 6 Department of Advanced Materials Science, University of Tokyo, Kashiwa, Chiba 277-8561 Japan

## Abstract

The structure of the superconducting gap in unconventional superconductors holds a key to understand the momentum-dependent pairing interactions. In superconducting FeSe, there have been controversial results reporting nodal and nodeless gap structures, raising a fundamental issue of pairing mechanisms of iron-based superconductivity. Here, by utilizing polarization-dependent laser-excited angle-resolved photoemission spectroscopy, we report a detailed momentum dependence of the gap in single- and multi-domain regions of orthorhombic FeSe crystals. We confirm that the superconducting gap has a twofold in-plane anisotropy, associated with the nematicity due to orbital ordering. In twinned regions, we clearly find finite gap minima near the vertices of the major axis of the elliptical zone-centered Fermi surface, indicating a nodeless state. In contrast, the single-domain gap drops steeply to zero in a narrow angle range, evidencing for nascent nodes. Such unusual node lifting in multi-domain regions can be explained by the nematicity-induced time-reversal symmetry breaking near the twin boundaries.

## Abstract (web summary)

The superconducting gap structure of FeSe remains a debated issue. Here, Hashimoto et al. report momentum dependence of the gap in single- and multi-domain regions of orthorhombic FeSe crystals, revealing an unusual node lifting of the gap structure in multi-domain regions.

## Introduction

Since the discovery of Fe-based superconductors^1^, they have been actively investigated to fully understand their superconducting (SC) mechanisms. Among the several kinds of Fe-based superconductors discovered so far, FeSe has the simplest crystal structure consisting only of SC layers^2^. After high-quality single crystals grown by the vapor transport method became available^3^, intrinsic properties of FeSe have attracted much attention. One of the noticeable properties of FeSe is its SC transition temperature (*T*_c_). While *T*_c_ of bulk FeSe is ∼10 K at ambient pressure^4^ and it increases to 37 K under applied pressure of 9 GPa^5^, it has been reported that superconductivity above 100 K is observed for single-layer FeSe grown on SrTiO_3_^6^. Another prominent feature of FeSe is its small Fermi energy (*ε*_F_), and thus, FeSe has been proposed to be in the crossover regime between weakly coupled Bardeen–Cooper–Schrieffer (BCS) and strongly coupled Bose–Einstein-condensate (BEC) limits^4^, where *ε*_F_ is comparable to the SC gap, as well as the bands around the Γ point of FeSe_1−*x*_Te_*x*_^7,8^.

The other features that may be directly related to the mechanism of superconductivity in FeSe are the existence of the structural transition from the tetragonal phase to the orthorhombic phase at *T*_s_ ∼ 90 K and the absence of the antiferromagnetic ordering^9^ unlike other Fe-based superconductors. It has been discussed based on the angle-resolved photoemission spectroscopy (ARPES) measurements^10–14^ that the structural transition is accompanied by orbital ordering. They reported that the splitting of the Fe 3*d*_*yz*_ and 3*d*_*zx*_ bands emerges at the M point around *T*_s_ and it increases as large as 50 meV. This large splitting has been considered to be too large for the crystal field splitting due to the structural transition, and thus regarded as the evidence of an electronically driven orbital ordering. On the other hand, however, several recent reports have interpreted this splitting as that of 3*d*_*yz*/*zx*_ and 3*d*_*xy*_^15,16^, or that due to the spin–orbit coupling^17^. At the Γ point, it has been confirmed that the 3*d*_*yz*_ and 3*d*_*zx*_ bands split, and one of the bands sinks below *E*_F_ and the other forms an elliptical hole Fermi surface (FS) by ARPES on the detwinned FeSe^18^. Similar inequivalent electron occupation of the Fe 3*d*_*yz*_ and 3*d*_*zx*_ orbitals has been observed by ARPES in Ba(Fe,Co)_2_As_2_^19^ and NaFeAs^20,21^. However, the interplay of this band splitting accompanied with the orbital ordering and superconductivity is still elusive. Revealing the SC gap anisotropy of the elliptical FS at the Γ point of FeSe should be crucial for understanding the role of the orbital ordering in superconductivity. In addition, the existence of line nodes in the SC gap of FeSe has been suggested from the thermal conductivity and scanning tunneling microscopy/spectroscopy (STM/STS) measurements^4^, although some reports suggest a fully gapped state without nodes^22–25^. Determination of the positions of the SC gap nodes is important for clarifying the SC mechanism.

In this paper, a study on the SC gap anisotropy of the zone-centered hole FS is presented based on the laser-excited ARPES measurements^26,27^ of single-crystal FeSe. We observe that the fourfold symmetry is significantly broken in the SC gap anisotropy, which is considered to be due to the orbital ordering, and find that while the SC gap node is not observed for multi-domain samples, it exists at the vertices of the major axis of the elliptical FS for single-domain samples. This can be attributed to breaking of time-reversal symmetry at the twin boundaries and our results reveal the effects of time-reversal symmetry breaking on the nodal SC gap anisotropy.

## Results

### Fermi surfaces and band dispersions of the twinned FeSe

**Fig. 1:**
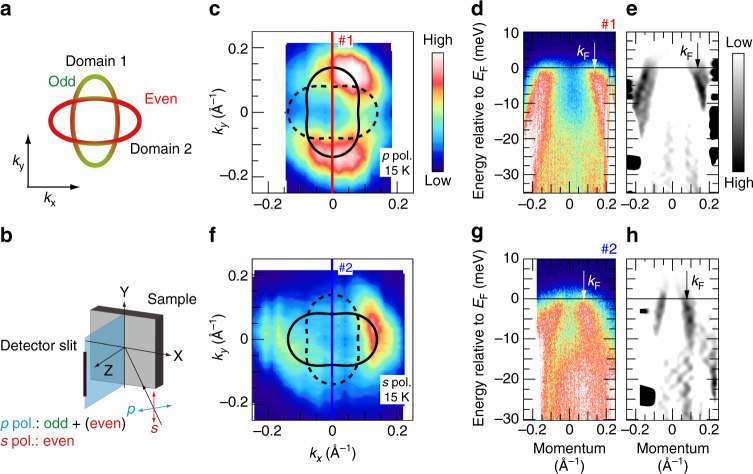
Electronic structure of the twinned FeSe at 15 K in the orbital ordered state. **a** Schematic FSs at the zone center. *x* and *y* are coordinates along the crystal axes of the orthorhombic setting. Due to the orbital ordering, two elliptical FSs are overlapped. Portions of the orbital contributions in those FSs are indicated by red and green for even and odd parity with respect to the mirror plane defined in **b**, respectively. **b** Experimental configuration. A mirror plane is defined to be parallel to the detector slit. Definition of *p*- and *s*-polarized light and sample axes is shown. **c** Plots of the ARPES intensity at *E*_F_ as a function of the two-dimensional wavevector measured with *p*-polarized light. The intensity is obtained by integrating the spectra within ±3 meV with respect to *E*_F_. The experimental FS (solid ellipse) and a duplicate rotated by 90° caused by twin domains (dashed ellipse) are shown. **d** ARPES intensity plot, **e** momentum second derivative of **d** at #1 in **c**. The arrow indicates a *k*_F_ position. **f**–**h** The same as **c**–**e** but taken with *s*-polarized light reflecting the other domain

Figure 1c and f shows the FS maps at the Brillouin-zone (BZ) center measured at 15 K (>*T*_c_), taken with *p*- and *s*-polarized incident light. Two FSs were observed and their shapes were twofold symmetric and elliptical. They are rotated to each other by 90° and elongated along the *k*_*y*_ and *k*_*x*_ directions, respectively. Photoemission intensity of the FS elongated along the *k*_*y*_ (*k*_*x*_) direction was higher for *p*-(*s*-) polarization. Below *T*_s_, the crystal symmetry is transformed from the tetragonal phase to the orthorhombic phase, and therefore, the formation of structural twins is generally inevitable. Observed polarization dependence can be explained by the orbital character of these two FSs due to twinning. Each Fe 3*d* orbital can be classified into even or odd parity with regard to the mirror plane which is parallel to the detector slit (see Supplementary Fig. 1 and Supplementary Note 1 for details). Due to the parity selection rule, *p*- (*s*-) polarized light predominantly observes orbital with odd (even) parity. Considering orbital components of the FS determined by the previous work^18^, the FS of two domains can be selectively observed by *p*- (*s*-) polarized light as shown in Fig. 1c, f. Figure 1d and g shows energy–momentum (*E*–*k*) images in cut #1 in Fig. 1c and cut #2 in Fig. 1f taken with *p*- and *s*-polarized light, respectively. Figure 1e and h are their momentum second derivatives. One can see that a hole band crosses the Fermi level (*E*_F_) at different Fermi wavevector *k*_F_ positions for each polarization. The different *k*_F_ positions of the observed bands correspond to those of the major and minor axes of the elliptical FS. The *k*_F_ positions are determined from momentum distribution curves (MDCs) at *E*_F_ (see Supplementary Fig. 6 and Supplementary Note 6 for details).

### SC gap structure of the twinned FeSe

**Fig. 2:**
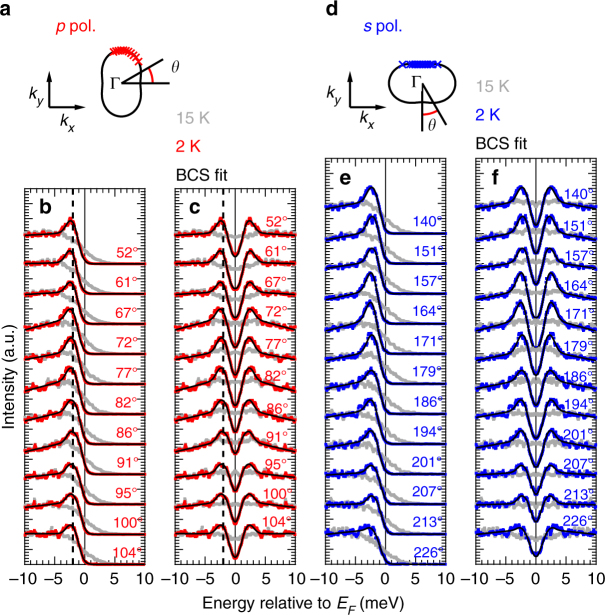
Superconducting gap anisotropy of twinned FeSe. **a** Definition of FS angle in case of *p*-polarized light. Red points indicate the *k*_F_ positions where energy distribution curves (EDCs) in **b** and **c** are taken. **b** EDCs at various *k*_F_ points along the FS at 15 K (gray) and 2 K (red). Black lines show the fits to the BCS fitting function. FS angle defined in **a** is shown for each EDC. **c** The same as **b** but symmetrized with respect to *E*_F_. **d**–**f** The same as **a**–**c** but taken with *s*-polarized light

Figure 2b and e shows the energy distribution curves (EDCs) at *k*_F_ below *T*_c_ and above *T*_c_ taken with *p*- and *s*-polarized light, respectively. Each EDC is identified with a FS angle *θ*, and the *k*_F_ positions are shown in Fig. 2a, d. To cancel out the effect of the Fermi–Dirac cutoff, the EDCs were symmetrized with respect to *E*_F_, and the results are shown in Fig. 2c, f. Sharp SC coherence peaks can be recognized very clearly in the spectra below *T*_c_. The vertical dashed line in Fig. 2c indicates the peak position of the EDC at *θ* = 91°, which is at the end of the major axis of the elliptical FS. The EDC at *θ* = 61°, for example, has a higher peak energy, indicating a finite SC gap anisotropy. Previous STM/STS measurements reported a two-peak structure^4^ indicating two SC gaps (Δ ∼ 2.5 and 3.5 meV). Current results correspond to the smaller gap, and the larger gap may exist at the zone corner. In order to quantify the SC gap sizes, we fitted the spectra to the BCS spectral function and the results are shown as solid lines (see Supplementary Note 7 for details of the fitting function). The observed spectra are well reproduced by the fitting function, indicating reliability of the obtained SC gap sizes.

### SC structure of single-domain FeSe

**Fig. 3:**
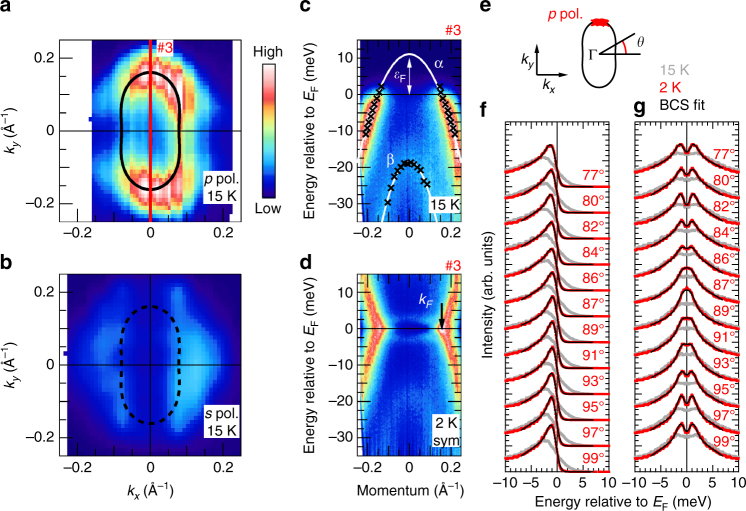
Superconducting gap anisotropy of single-domain FeSe without uniaxial tensile strain. **a** Plots of the ARPES intensity at *E*_F_ of FeSe as a function of the two-dimensional wavevector measured with *p*-polarized light. The intensity is obtained by integrating the spectra within 3 meV with respect to *E*_F_. Black ellipse indicates the experimental FS. **b** The same as **a** but measured with *s*-polarized light. **c** ARPES intensity plot above *T*_c_ (15 K) at the momentum line shown in **a**, taken with *p*-polarized light. Black markers represent the experimental band dispersion determined from momentum distribution curves and energy distribution curves (EDCs) for the *α* and *β* band. The Fermi energy *ε*_F_ is shown for the *α* band. **d** The same as **c** but taken below *T*_c_ (2 K) and the data are symmetrized with respect to *E*_F_. The black arrow indicates the *k*_F_ position. **e** Definition of FS angle. The red points indicate the *k*_F_ values where EDCs in **f** and **g** are taken. **f** EDCs at different *k*_F_ values along the FS at 15 K (gray) and 2 K (red). Black lines show the fits to the BCS fitting function. The FS angles defined in **e** are shown for each EDC. **g** Same as **f** but symmetrized at *E*_F_

Figure 3 shows the results for another sample, which has a different intensity ratio between two polarizations from Fig. 1. The observed FSs are shown in Fig. 3a, b. The intensity of the FS observed by *s*-polarized light is much weaker compared to that by *p*-polarized light, and this intensity difference is similar to that of the detwinned sample (Supplementary Fig. 2 and Supplementary Note 2). By contrast, Fig. 1c, f shows similar intensity of the FS between *s*- and *p*-polarized light. Considering this difference, the results shown in Fig. 3 can be interpreted as observation of the single-domain region, although any intentional uniaxial tensile strain was not applied to the sample. This is probably owing to the small laser spot size (∼200 μm) and the large domain size of the cleaved surface (see Supplementary Fig. 5 and Supplementary Note 5 for the position dependence of the spectra). Similar results for the observation of the single-domain region have been reported for FeSe by Raman scattering^28^ and ARPES^29^.

The clear image in Fig. 3c enables a discussion about the band dispersions. The value of *k*_*z*_ for the present results obtained with 7-eV laser can be estimated from the position of the *β* band, which lies below *E*_F_ as shown in Fig. 3c. The top of the *β* band is located at ∼18 meV below *E*_F_. Watson et al. have reported that the top of the *β* band is located at ∼25 and 16 meV below *E*_F_ for the Γ and Z point, respectively^12^. Therefore, *k*_*z*_ for 7 eV can be estimated to be closer to the Z point than the Γ point according to the results of Watson et al. As mentioned above, FeSe has been suggested to be in a BCS–BEC crossover regime^4^. The present result shows *ε*_F_ ∼ 10 meV for the *α* band (Fig. 3c). This corresponds to $\Delta /\varepsilon _{\mathrm{F}} \sim$ 0.2 for the hole band at the BZ center. This is almost consistent with the previous report of $\Delta /\varepsilon _{\mathrm{F}} \sim$ 0.3 from the STM/STS measurements^4^, which is smaller than the criteria of the BCS–BEC crossover regime, and should be an evidence that our results are reasonable and reliable. The electron band at the BZ corner might have $\Delta /\varepsilon _{\mathrm{F}} \sim$ 1 and satisfy this criterion, as suggested from the STS/STM measurements^4^.

Figure 3d shows the *E–k* image below *T*_c_ (2 K) of cut #3 indicated in Fig. 3a, symmetrized with respect to *E*_F_. This shows no detectable gap, which may demonstrate the existence of SC gap nodes. Figure 3f and g shows the EDCs at *k*_F_ and symmetrized EDCs with respect to *E*_F_, respectively, taken with *p*-polarized light. Each EDC is identified with a FS angle *θ*, and the momentum positions in the FS are shown in Fig. 3e. Some of the symmetrized EDCs above *T*_c_ (15 K) seem to have a pseudogap. This might correspond to the preformed Cooper pairing associated with the BCS–BEC crossover regime reported by Kasahara et al^30^. They reported that the experimental signatures of the preformed Cooper pairing were observed below 20 K. It is clear from the spectra that the SC gap becomes smaller as *θ* reaches to 90°. Furthermore, the spectra around *θ* = 90° show an undetectable gap, and thus, nodes may exist around *θ* = 90°. On the other hand, since the experimental observation limit is estimated to be ∼0.2 meV, the SC gap minimum is at least smaller than 0.2 meV. The BCS spectra fitting is shown in Fig. 3f, g as solid lines.

**Fig. 4:**
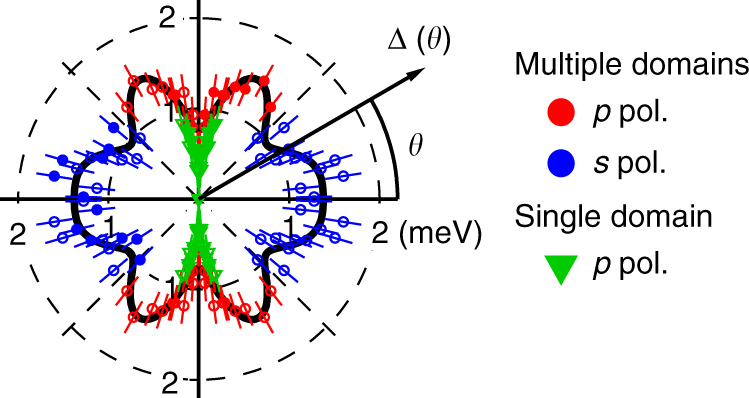
Superconducting gap anisotropy of the elliptical Fermi surface. Results of the multi-domain sample with *p*- (red circle) and *s*- (blue circle) polarized light are shown together, considering that each polarization predominantly probes different domains. Results of the single-domain sample (green triangle) are also shown together. Solid symbols are obtained from BCS spectra fitting and open symbols are symmetrized by taking into account the orthorhombic crystal symmetry. Error bars are determined by the systematic and statistical error of the calibrated *E*_F_ positions, as explained in Supplementary Note 8. The black curve is fitting to the observed anisotropy of the multi-domain sample, considering the twofold orthorhombic symmetry. The fitting function is $\Delta (\theta ) = |A + B\;{\rm{cos}}(2\theta ) + C\;{\rm{cos}}(4\theta ) + D\;{\rm{cos}}(6\theta ) + E\;{\rm{cos}}(8\theta )|$

Figure 4 shows the obtained SC gap anisotropy. The results from the multi- and single-domain samples are shown together. For the multi-domain samples, the results with *p*- and *s*-polarized light are shown together, considering that each polarization shows a higher intensity for the domain rotated to each other by 90°. We fitted the results of the multi-domain samples to the following twofold symmetric formula of the summation of harmonic series:

1 $$\begin{array}{*{20}{l}} {\Delta (\theta ) = |A + B\;{\rm{cos}}(2\theta ) + C\;{\rm{cos}}(4\theta )} \hfill \\ { + D\;{\rm{cos}}(6\theta ) + E\;{\rm{cos}}(8\theta )|,} \hfill \end{array}$$

and the obtained fitting parameters were *A* = 1.19 meV, *B* = 0.079 meV, *C* = 0.02 meV, *D* = 0.228 meV, and *E* = –0.141 meV.

The observed SC gap anisotropy shows two major characteristics. First, it shows twofold symmetry. The orbital ordering makes the electronic structure twofold symmetric, and the SC gap anisotropy follows the symmetry. The SC gap at *θ* = 90° shows a minimum, while that at *θ* = 0° shows a maximum. This clearly demonstrates the breaking of the fourfold symmetry of the SC gap anisotropy. Fitting of the SC gap anisotropy also shows the breaking of the fourfold symmetry as well. Second, the observed SC gap anisotropy shows sharp anisotropy around *θ* = 90°. The gap shows a sharp drop toward *θ* = 90°. In contrast, the anisotropy becomes very weak around *θ* = 180°. These two observations show the necessity for considering the fourfold symmetry breaking due to the orbital ordering when one pursues the mechanism of superconductivity in FeSe. Additionally, there are local minima around *θ* = ±45° and ± 135°. The breaking of fourfold symmetry in SC gap anisotropy is basically consistent with very recent reports of ARPES on a similar compound FeSe_0.93_S_0.07_^31^, and Bogoliubov quasiparticle interference (BQPI) on FeSe^32^. Theoretically, twofold symmetry of SC gap anisotropy is explained based on spin fluctuations^33,34^, cooperation between spin and orbital fluctuations^35^, competition between nematic order and superconductivity^36^, or orbital nematic fluctuations^37^.

**Fig. 5:**
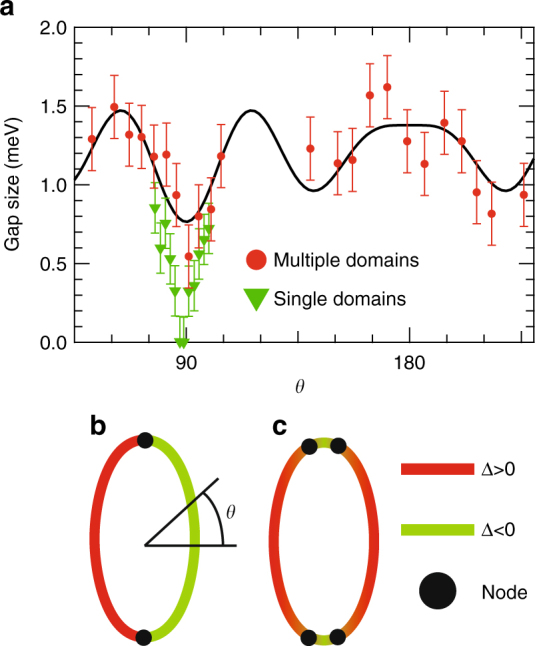
Superconducting gap anisotropy of multi- and single-domain FeSe. **a** Superconducting gap anisotropy of the elliptical FS, shown in Cartesian coordinates. Results of the multi-domain samples (red circle) and single-domain samples (green triangle) are shown together. Error bars are determined by the systematic and statistical error of the calibrated *E*_F_ positions, as explained in Supplementary Note 8. The black curve is fitting to the observed anisotropy of the multi-domain samples, considering the twofold orthorhombic symmetry. The fitting function is $\Delta (\theta ) = |A + B\;{\rm{cos}}(2\theta ) + C\;{\rm{cos}}(4\theta ) + D\;{\rm{cos}}(6\theta ) + E\;{\rm{cos}}(8\theta )|$. Schematic description of gap signs on the Γ-centered FS assuming **b** a single node and **c** two nodes. The red (green) line indicates Δ > 0 (<0), and the black circles are the positions of nodes. Definition of *θ* is shown

The observed SC gap anisotropy summarized in Fig. 5a shows a considerable difference between the multi- and single-domain samples. The results for the multi-domain samples show finite gaps at any *θ*, while those for the single-domain samples show an undetectable gap around *θ* = 90°. The difference of the SC gap size between the multi- and single-domain samples away from *θ* = 90° becomes small and is almost within error bars at *θ* = 80° and 100°.

## Discussion

It is not likely that this different gap anisotropy is caused by the difference of disorder level among the pieces of samples. Teknowijoyo et al. have reported from the London penetration depth measurements that the gap minimum increases by ∼0.05 meV after introducing point-like disorder by electron irradiation^24^, when the created Frenkel pairs of interstitial vacancies are estimated to be ∼0.05% per Fe and per Se (0.1% total pairs per formula). For our single crystals, the number of impurities and defects was confirmed to be <0.05% per Fe by scanning tunneling microscope topography^4,38^, and thus, the increase of the gap minimum due to impurities and defects is expected to be less than ∼0.05 meV, which is much smaller than the difference of the gap minimum at *θ* = 90° between the multi- and single-domain samples (∼0.5 meV).

Alternatively, SC gap anisotropy could be affected by the existence of twin boundaries. According to Watashige et al., it has been suggested that twin boundaries induce a twist of the order parameter, and accordingly, time-reversal symmetry is broken and a fully gapped state is observed over a distance several times larger than the coherence length near twin boundaries^38^. Because there should exist many twin boundaries within the laser spot for the multi-domain samples, the SC gap anisotropy for the multi-domain samples could reflect a fully gapped state due to time-reversal symmetry breaking. This could settle a contradiction between the different results for the thermal conductivity measurements. While Kasahara et al. observed a large $\kappa _0/T$ (a residual linear term in the thermal conductivity as *T* → 0 K) and suggested line nodes in the SC gap^4^, Bourgeois-Hope et al. observed a very small $\kappa _0/T$^22^, although a residual resistivity is not significantly different between these two studies. This difference might be due to the density of twin boundaries. Moreover, the difference of the SC gap anisotropy between the multi- and single-domain samples around *θ* = 90° is consistent with the theoretically calculated node disappearance due to time-reversal symmetry breaking^39^. Therefore, the difference of the SC gap anisotropy between the multi- and single-domain samples is considered as the effect of time-reversal symmetry breaking near twin boundaries.

According to the SC gap determined from BQPI^32^, significantly anisotropic gap has been suggested for the zone-centered hole FS. Although the gap nodes were not observed by BQPI, since its reason might be due to the finite scanned area for the Fourier transform, our results should be totally consistent with the gap anisotropy determined from BQPI. If a single node is assumed at the vertex, this means that a sign change occurs at each vertex of the major axis (*θ* = ±90°) as schematically shown in Fig. 5b. Although the signed values of the SC gap should become more continuous at *θ* = ±90° in this case, the SC gap symmetry is considered to be a* p*-wave. This would be difficult to expect because there is no theoretical argument for *p*-wave pairing in this system and this seems inconsistent with the temperature dependence of the upper critical field^40,41^. Thus, two nodes are assumed to exist at each vertex of the major axis (*θ* = ±90°) as shown in Fig. 5c, similar to KFe_2_As_2_, which shows an octet-line node structure where two nodes exist within the narrow FS angle range^26^. In this case, the sign changes occur twice around the vertices and the sign of the gap is consistent with the *s*-wave symmetry.

## Methods

### Sample preparation

High-quality single crystals were grown by the chemical vapor transport method using KCl/AlCl_3_ as transport agent as described in ref. ^3^.

### Laser-ARPES measurements

ARPES data were collected using a laser-ARPES apparatus at ISSP with 6.994 eV, sixth harmonics of Nd:YVO_4_ quasi-continuous-wave (repetition rate = 960 MHz) laser, and VG-Scienta HR8000 electron analyzer as described in ref. ^26^. This apparatus achieves a maximum energy resolution of 70 μeV and the lowest cooling temperature of 1.5 K, which enables a direct measurement of the SC gap of FeSe. The overall energy resolution was set to ∼1.2 meV and the angular resolution was 0.1°. The Fermi edge of an evaporated gold film was measured to calibrate *E*_F_ energy positions. The error bars of the SC gap size were determined from the stability of *E*_F_ position, and evaluated to be 200 μeV. More details for the accuracy of the measured gap size were described in the previous reports^26,42^. Polarization of the incident excitation laser was adjusted using half-wave (*λ*/2) and quarter-wave (*λ*/4) plates. Samples were cleaved in situ under ultrahigh vacuum and measurements were carried out at pressures better than 5 × 10^−11^ Torr. The measurements were limited to the hole FS at the zone center due to the relatively low excitation energy of 6.994 eV, with which the momentum around the electron FS at the zone corner cannot be accessed.

### Data availability

The data supporting the findings of this study are available from the corresponding author on request.

## Electronic supplementary material

Supplementary Information

## References

[1] Kamihara Y, Watanabe T, Hirano M, Hosono H (2008). Iron-based layered superconductor La[O_1−*x*_F_*x*_]FeAs (x=0.05-0.12) with *T*_*c*_=26 K. J. Am. Chem. Soc..

[2] Hsu FC (2008). Superconductivity in the PbO-type structure *α*-FeSe. Proc. Natl Acad. Sci. USA.

[3] Böhmer AE (2013). Lack of coupling between superconductivity and orthorhombic distortion in stoichiometric single-crystalline FeSe. Phys. Rev. B.

[4] Kasahara S (2014). Field-induced superconducting phase of FeSe in the BCS-BEC cross-over. Proc. Natl Acad. Sci. USA.

[5] Medvedev S (2009). Electronic and magnetic phase diagram of *β*-Fe_1.01_Se with superconductivity at 36.7K under pressure. Nat. Mater..

[6] Ge JF (2014). Superconductivity above 100K in single-layer FeSe films on doped SrTiO_3_. Nat. Mater..

[7] Lubashevsky Y, Lahoud E, Chashka K, Podolsky D, Kanigel A (2012). Shallow pockets and very strong coupling superconductivity in FeSe_*x*_Te_1−*x*_. Nat. Phys..

[8] Okazaki K (2014). Superconductivity in an electron band just above the Fermi level: possible route to BCS-BEC superconductivity. Sci. Rep..

[9] McQueen TM (2009). Tetragonal-to-orthorhombic structural phase transition at 90 K in the superconductor Fe_1.01_Se. Phys. Rev. Lett..

[10] Shimojima T (2014). Lifting of *xz*/*yz* orbital degeneracy at the structural transition in detwinned FeSe. Phys. Rev. B.

[11] Zhang P (2015). Observation of two distinct *d*_*xz*_/*d*_*yz*_ band splittings in FeSe. Phys. Rev. B.

[12] Watson MD (2015). Emergence of the nematic electronic state in FeSe. Phys. Rev. B.

[13] Nakayama K (2014). Reconstruction of band structure induced by electronic nematicity in an fese superconductor. Phys. Rev. Lett..

[14] Fanfarillo L (2016). Orbital-dependent Fermi surface shrinking as a fingerprint of nematicity in FeSe. Phys. Rev. B.

[15] Watson MD (2016). Evidence for unidirectional nematic bond ordering in FeSe. Phys. Rev. B.

[16] Fedorov A (2016). Effect of nematic ordering on electronic structure of FeSe. Sci. Rep..

[17] Borisenko SV (2016). Direct observation of spin-orbit coupling in iron-based superconductors. Nat. Phys..

[18] Suzuki Y (2015). Momentum-dependent sign inversion of orbital order in superconducting FeSe. Phys. Rev. B.

[19] Yi M (2011). Symmetry-breaking orbital anisotropy observed for detwinned Ba(Fe_1−*x*_Co_*x*_)_2_As_2_ above the spin density wave transition. Proc. Natl Acad. Sci. USA.

[20] Zhang Y (2012). Symmetry breaking via orbital-dependent reconstruction of electronic structure in detwinned NaFeAs. Phys. Rev. B.

[21] Yi M (2012). Electronic reconstruction through the structural and magnetic transitions in detwinned NaFeAs. New J. Phys..

[22] Bourgeois-Hope P (2016). Thermal conductivity of the iron-based superconductor FeSe: nodeless gap with a strong two-band character. Phys. Rev. Lett..

[23] Jiao L (2017). Superconducting gap structure of FeSe. Sci. Rep..

[24] Teknowijoyo S (2016). Enhancement of superconducting transition temperature by pointlike disorder and anisotropic energy gap in FeSe single crystals. Phys. Rev. B.

[25] Li M (2016). Superfluid density and microwave conductivity of FeSe superconductor: ultra-long-lived quasiparticles and extended s -wave energy gap. New J. Phys..

[26] Okazaki K (2012). Octet-line node structure of superconducting order parameter in KFe_2_As_2_. Science.

[27] Shimojima T, Okazaki K, Shin S (2015). Low-temperature and high-energy-resolution laser photoemission spectroscopy. J. Phys. Soc. Jpn.

[28] Hu Y (2016). Nematic magnetoelastic effect contrasted between Ba(Fe_1−*x*_Co_*x*_)_2_As_2_ and FeSe. Phys. Rev. B.

[29] Watson MD, Haghighirad AA, Rhodes LC, Hoesch M, Kim TK (2017). Electronic anisotropies revealed by detwinned angle-resolved photo-emission spectroscopy measurements of FeSe. New J. Phys..

[30] Kasahara S (2016). Giant superconducting fluctuations in the compensated semimetal FeSe at the BCS-BEC crossover. Nat. Commun..

[31] Xu HC (2016). Highly anisotropic and twofold symmetric superconducting gap in nematically ordered FeSe_0.93_S_0.07_. Phys. Rev. Lett..

[32] Sprau PO (2017). Discovery of orbital-selective Cooper pairing in FeSe. Science.

[33] Kreisel A (2017). Orbital selective pairing and gap structures of iron-based superconductors. Phys. Rev. B.

[34] She, J.-H., Lawler, M. J. & Kim, E.-A. Mechanism for nematic superconductivity in FeSe. Preprint at http://arxiv.org/abs/1701.07813 (2017).

[35] Yamakawa Y, Kontani H (2017). Nematicity, magnetism, and superconductivity in FeSe under pressure: Unified explanation based on the self-consistent vertex correction theory. Phys. Rev. B.

[36] Mishra V, Hirschfeld PJ (2016). Effect of disorder on the competition between nematic and superconducting order in FeSe. New J. Phys..

[37] Agatsuma T, Yamase H (2016). Structure of the pairing gap from orbital nematic fluctuations. Phys. Rev. B.

[38] Watashige T (2015). Evidence for time-reversal symmetry breaking of the superconducting state near twin-boundary interfaces in FeSe revealed by scanning tunneling spectroscopy. Phys. Rev. X.

[39] Maiti S, Chubukov AV (2013). *s*+*is* state with broken time-reversal symmetry in Fe-based superconductors. Phys. Rev. B.

[40] Terashima T (2014). Anomalous Fermi surface in FeSe seen by Shubnikov de Haas oscillation measurements. Phys. Rev. B.

[41] Vedeneev SI, Piot BA, Maude DK, Sadakov AV (2013). Temperature dependence of the upper critical field of FeSe single crystals. Phys. Rev. B.

[42] Ota Y (2017). Unconventional superconductivity in the Bis_2_-based layered superconductor NdO_0.71_F_0.29_Bis_2_. Phys. Rev. Lett..

