# Supplementary material for: Superconducting gap anisotropy sensitive to nematic domains in FeSe
Source: Nat Commun. 2018 Jan 18;9:282. doi: 10.1038/s41467-017-02739-y (PMC5773685; doi:10.1038/s41467-017-02739-y)
Supplement: Supplementary file 1 — Supplementary Information [file 41467_2017_2739_MOESM1_ESM.pdf]

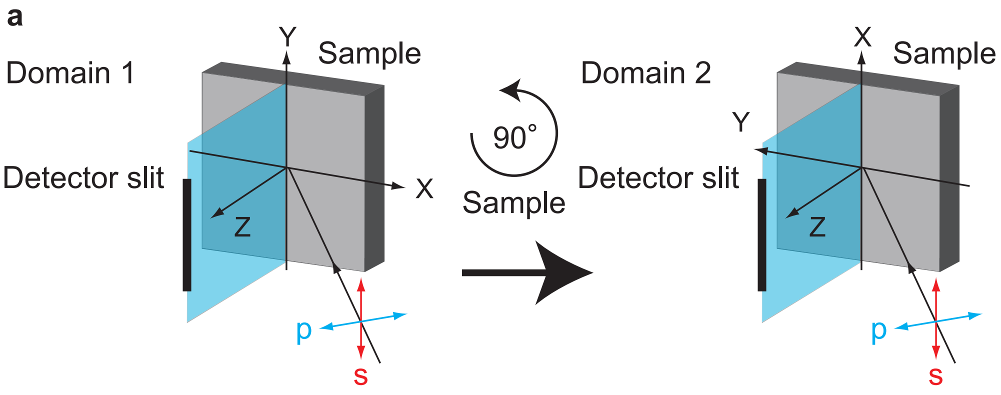

p pol.: odd + (even)

s pol.: even

b

	Domain 1	Domain 2
XZ	odd	even
YZ	even	odd

Supplementary Figure 1. The experimental configuration. (a) Schematic illustration of experimental configuration for two domains below the structural transition temperature (see Supplementary Note 1 for detail). Axis of each orbital is fixed to sample. (b) Parity for each orbital in each domain.

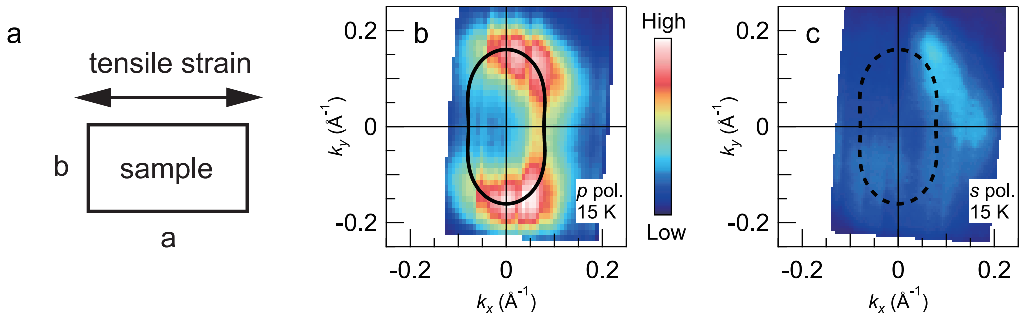

Supplementary Figure 2. The Fermi surface map for the detwinned sample. (a) Schematic illustration of the experimental configuration. Uniaxial tensile strain was induced to the sample to align the direction of the orthorhombic crystal (a>b). (b) Plots of the ARPES intensity at $E_{\rm F}$ for the detwinned FeSe as a function of the two-dimensional wavevector measured with p-polarized light. The intensity is obtained by integrating the spectra within 3 meV with respect to $E_{\rm F}$. The black ellipse indicates the experimental FS. (c) The same as (b) but taken with s-polarized light. The black dotted ellipse indicates absence of the FS.

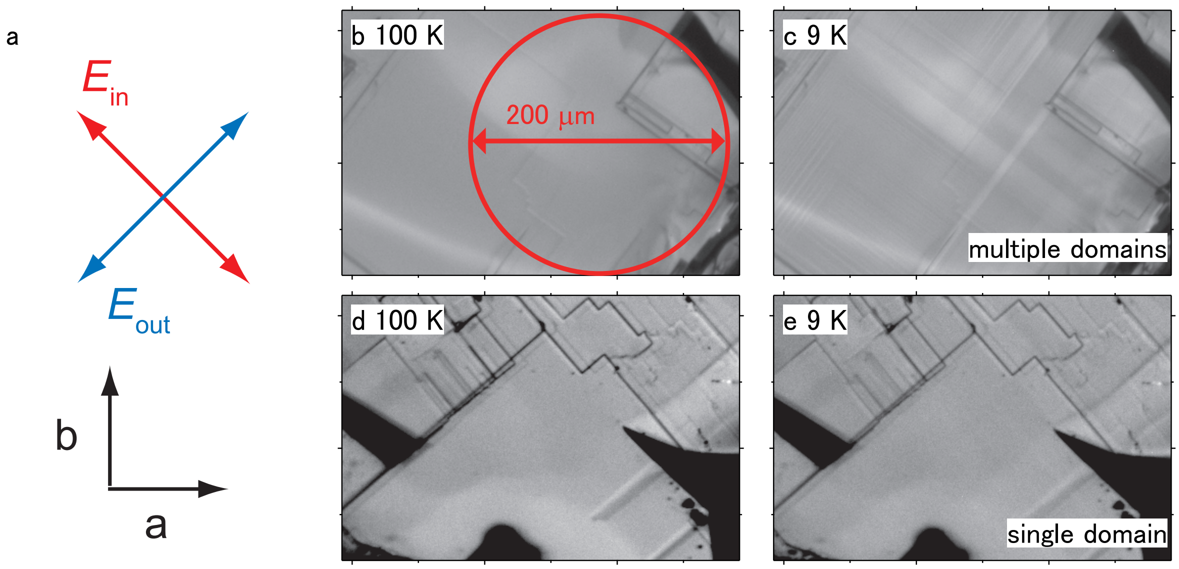

Supplementary Figure 3. Polarization microscope images of sample surfaces. (a) Direction of polarizations for the incident and detected light with respect to the orthorhombic crystal axes. Polarizations of the incident and detected light (E_{in} and E_{out}) are indicated by red and blue arrows, respectively. (b), (c) Images of the multi-domain sample taken by polarization microscope at 100 K (b) and 9 K (c), respectively. The red circle corresponds to the spot size of the incident laser ($\sim$ 200 μm). Diagonal line structures in (c) correspond to twin boundaries. (d), (e) Images of the single-domain sample at 100 K (d) and 9 K (e), respectively.

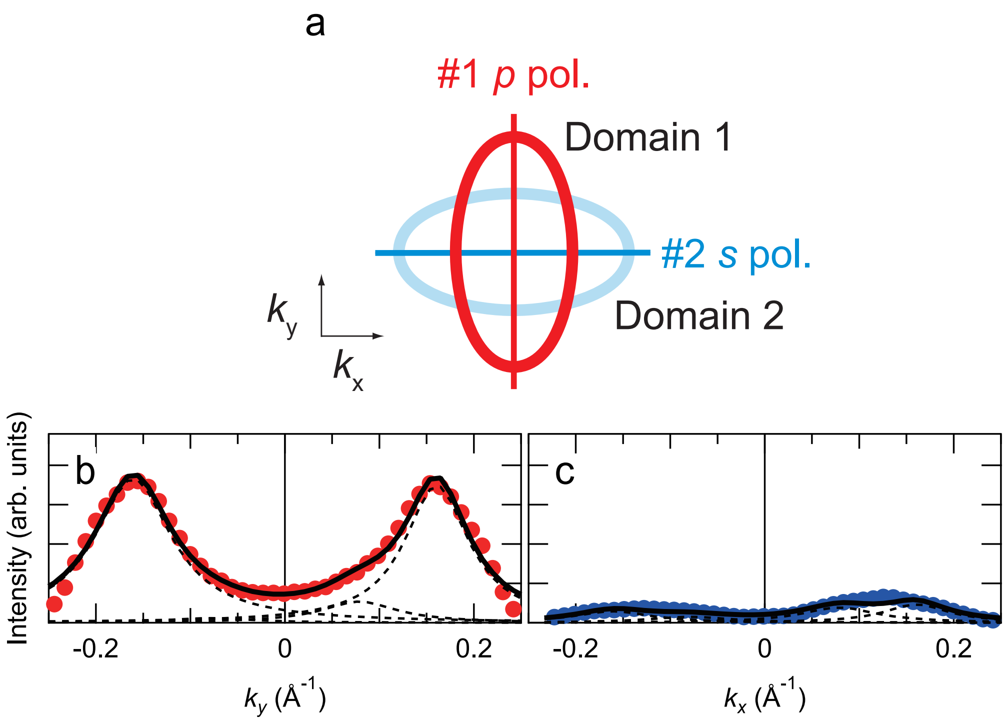

Supplementary Figure 4. Estimation of the percentages of two domains in the single-domain sample. (a) Schematic image of the FS for the single-domain sample. The red and blue ellipses indicate the FS of the domain 1 and domain 2, respectively. (b) Momentum distribution curve (MDC) taken with p-polarized light along the k_y direction. The solid black line is the fitting function composed of four Lorentzians. The dashed black lines correspond to each component of the Lorentzians. (c) MDC taken with s-polarized light along the k_x direction.

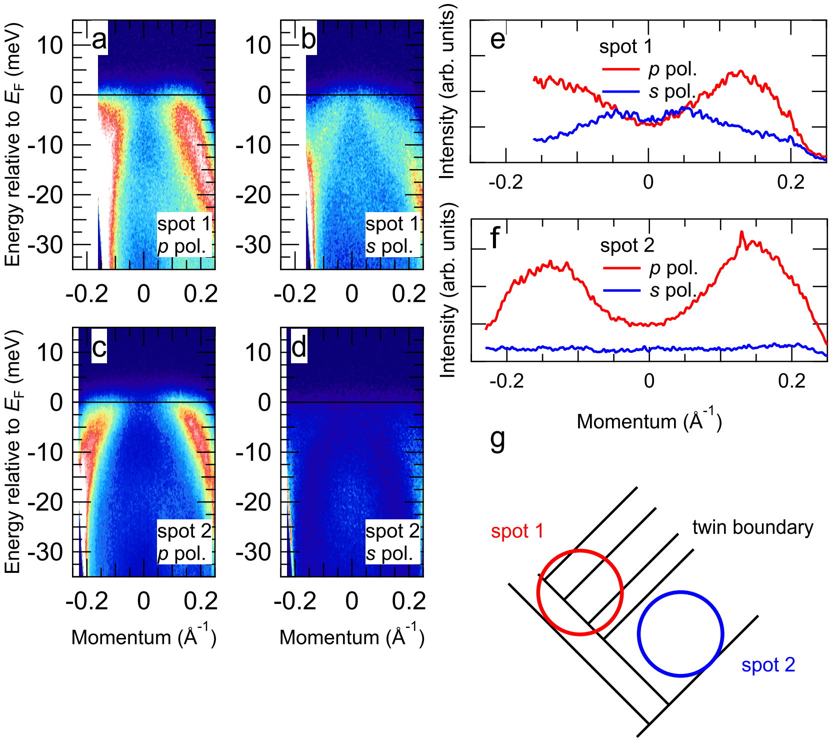

Supplementary Figure 5. Position and polarization dependence of the ARPES spectra. (a), (b) ARPES intensity plots along the k_y direction taken at the spot 1 with p- and s- polarized light, respectively. (c), (d) the same as (a) and (b) but taken at the spot 2, which is 400 μm away from the spot 1. (e), (f) Momentum distribution curves taken with p- and s- polarized light at the spot 1 and 2, respectively. The energy integration window was $\pm$ 3 meV with respect to E_F. (g) Schematic image of the sample surface and the spots 1 and 2. The solid lines indicate twin boundaries.

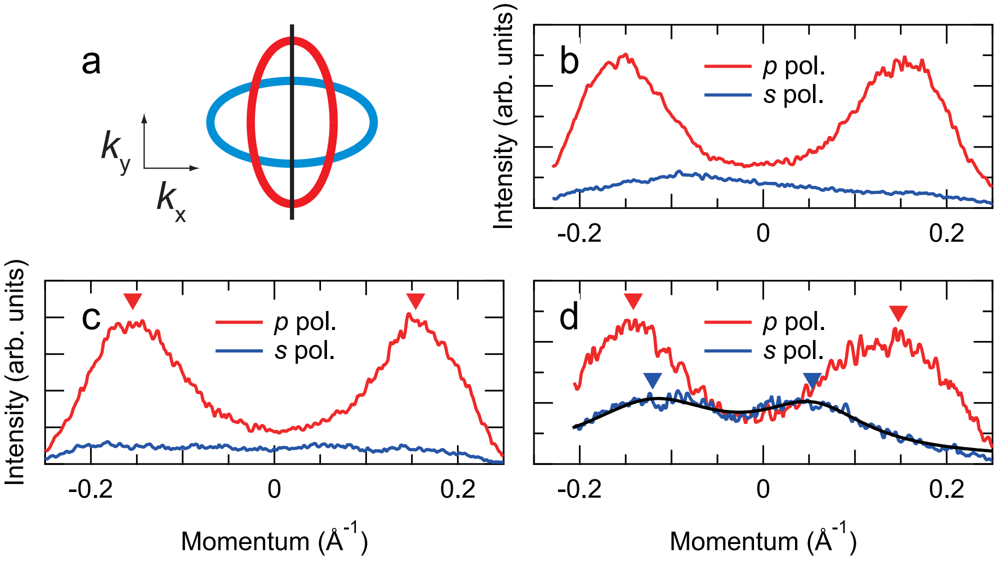

Supplementary Figure 6. Determination of the Fermi wave vector positions. (a) Schematic description of the FS at the zone center for the domain 1 (red) and the domain 2 (blue). The black line indicates the momentum cut for the momentum distribution curves (MDCs). (b)-(d) MDCs at E_{F} of the detwinned sample (b), single-domain sample (c), and multiple-domain sample (d), respectively. The energy integration window for these MDCs is $\pm$ 3 meV with respect to E_{F}. The red and blue triangles indicate the k_{F} positions along the major and minor axes of the elliptical FS determined from the MDCs taken with p- and s-polarized light, respectively. The black curve in (d) is the fitting result for the MDC taken with s-polarized light.

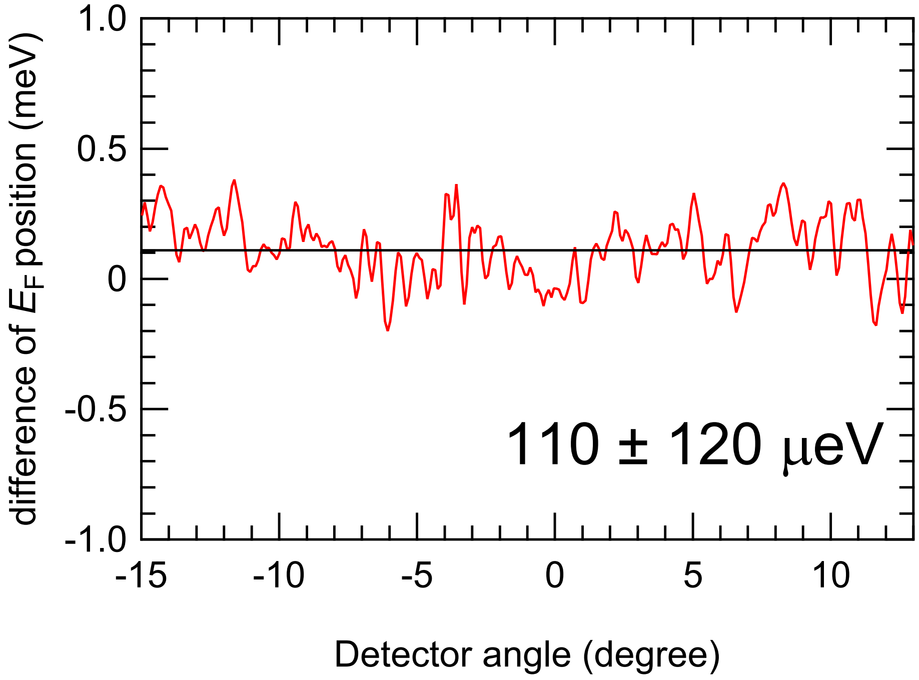

Supplementary Figure 7. Fermi level positions of the gold film evaporated on the sample holder calibrated by the reference. The E_{F} position at each detector channel was determined by fitting the energy distribution curve at each channel to the Gaussian-broadened Fermi Dirac function.

Supplementary Note 1. Experimental configuration for polarization-dependent Angle resolved photoemission spectroscopy measurements

The parity selection rule enables orbital-selective observations in Angle resolved photoemission spectroscopy (ARPES) [1]. The experimental configuration is shown in Supplementary Figure 1. We select $yz(zx)$ plane as a mirror plane for the domain 1(2), and parity is defined with regard to this mirror plane. Each Fe $3d$ orbital can be classified to even and odd parity. In case of the domain 1, d_{zx} orbital has odd and d_{yz} orbital has even parity. When the detector slit is in the mirror plane (an analyzer normal configuration), a photoelectron which enters the detector slit has even parity. In case of the analyzer normal configuration, the parity selection rule can be described as that the transition matrix elements are finite only when the initial state and incident light has the same parity [1]. p-polarized light which has odd parity portion probes d_{zx} orbital, while s-polarized light which has only even parity probes only d_{yz} orbital. The domain 2 is rotated to 90 degrees from the domain 1, and the parity selection rule for d_{zx} and d_{yz} becomes opposite (note that each axis is fixed to the sample).

Supplementary Note 2. Fermi surface map of the detwinned sample

Supplementary Figure 2 shows the results for the detwinned sample. Detwinning is the alignment of the direction of the orthorhombic crystal by applying uniaxial tensile strain to the sample [2–4]. Absence of the elliptical Fermi surface (FS) for s-polarized light, which shows a striking difference from the results for the twinned sample (Fig. 1 of the main text), confirms that the sample was successfully detwinned. We tried to obtain the gap structure of the detwinned samples, but unfortunately, we found that precise measurements to obtain the gap structure within the error bar of 200 μeV were very difficult with the detwinning device. This is probably because the stability of the Fermi level (E_{F}) position becomes worse and the lowest achievable temperature becomes slightly higher due to the worse electrical and thermal conductance of the sample holder attached with the detwinning device.

Supplementary Note 3. Domain structures observed by polarization microscope

We tried to observe domain structures by polarization microscope [5]. Using this technique, twin boundaries can be visualized due to the difference of reflectivity along orthorhombic crystal axes. In the multi-domain sample, line structures were observed in the orthorhombic phase as shown Supplementary Figure 3c, and they are tilted by 45° to the orthorhombic crystal axes. Above the structural transition temperature ($T_s \sim 90$ K), the crystal becomes tetragonal, and these line structures were not observed as shown in Supplementary Figure 3b. Therefore, these structures correspond to twin boundaries as reported by Tanatar *et al.* [6]). According to the scanning tunneling microscopy measurements, the fully gapped state has been observed in much wider region than the width of twin boundaries [7]. Therefore, if many small twin boundaries, though the domains smaller than 1 μm is difficult to observed by optical microscope, exist in the multi-domain sample as shown in Supplementary Figure 3c, it can be expected that no superconducting gap node is observed by ARPES. On the other hand, no line structure was observed in the single domain sample as shown in Supplementary Figure 3e.

Supplementary Note 4. Estimation of the percentages of two domains in the single-domain sample

Even in the single-domain sample, the percentage of one domain is not 100 %. We have estimated the percentages of the domain 1 and domain 2 (defined in Supplementary Figure 1) from the fitting of the momentum distribution curves (MDCs) along the vertical and horizontal cut for the single-domain sample as shown in Supplementary Figure 4. Although the matrix element for each polarization is necessary for more precise estimation, it can be roughly estimated by assuming that the matrix elements of d_{zx} orbital for the odd portion of p-polarization and d_{yz} orbital for s-polarization are equal. From the ratio of intensity corresponding to the domain 1 and domain 2, the percentage of the domain 2 was estimated to be ~ 14 %.

Supplementary Note 5. Distribution of single- and multi-domain regions on one cleaved surface.

Supplementary Figure 5 shows the position and polarization dependence of ARPES spectra. At the spot 1 schematically drawn in Supplementary Figure 5g, the ARPES images taken with p- and s-polarized light show different dispersions as shown in Supplementary Figures 5a and S5b, respectively, similar to the results of the multi-domain sample shown in Figs. 1d and 1g of the main text. On the other hand, at the spot 2, the ARPES intensity is very weak for s-polarized light compared to p-polarized light as shown in Supplementary Figures 5c and 5d. The intensity of the MDC taken at the spot 2 with s-polarized is very weak compared to the others and has no peak as shown in Supplementary Figures 5e and 5f. These differences demonstrate that both of multi- and single-domain regions exist on one cleaved surface.

Supplementary Note 6. Determination of the Fermi wave vector positions

The precise determination of the Fermi wave vector (k_{F}) positions is quite important for the precise determination of the superconducting gap. We determined the k_{F} positions from the peak positions of the MDCs at E_{F} or fitting of the MDCs to Lorentzians as shown in Supplementary Figure 6. Supplementary Figure 6 shows the polarization-dependent MDCs for the detwinned, single-domain, and multiple-domain samples. For the MDCs taken with p-polarized light, the MDC peaks could be recognized very clearly, and thus we determined k_{F} from the peak positions. The reason why no peak was observed for the MDCs of the detwinned and single domain samples taken with s- polarized light is because the percentage of the domain 2 (defined in Supplementary Figure 1), of which FS should be observed like the blue ellipse described in Supplementary Figure 6a, is very small for these samples. On the other hand, for the MDC of the multiple-domain sample taken with s-polarized light, for which the MDC peaks corresponding to the FS described by the blue ellipse in Supplementary Figure 6a should be observed, two peaks are close to each other, we determined the k_{F} positions from the fitting to two Lorentzians. We tried to find the other type of the single-domain sample, domain 2, of which FS should be observed like the blue ellipse described in Supplementary Figure 6a, but it could not be observed.

Supplementary Note 7. Determination of the superconducting gap size by fitting to the Bardeen-Cooper-Schrieffer spectral function

The superconducting (SC) gap size was obtained by fitting to the Bardeen-Cooper-Schrieffer (BCS) spectral function [8, 9]. The BCS spectral function is given by

$$A(k,\omega) = \frac{1}{\pi}\left\{ \frac{|u_k|^2\Gamma}{(\omega - E_k)^2 + \Gamma^2} + \frac{|v_k|^2\Gamma}{(\omega + E_k)^2 + \Gamma^2} \right\}, \quad (1)$$

where $|u_k|^2$ and $|v_k|^2$ are the coherence factors, Γ is the broadening factor due to the quasiparticle lifetime, E_k is the band dispersion of Bogoliubov quasiparticles. From the normal-state band dispersion ε_k and the SC gap size $\Delta(k)$, E_k is given by

$$E_k = \left[\varepsilon_k^2 + |\Delta(k)|^2\right]^{1/2}. \quad (2)$$

In order to obtain a fitting function, the BCS spectral function is multiplied by a Fermi Dirac function after the integral (Shirley-type) background is added, and convoluted with a Gaussian corresponding to the experimental energy resolution. Since the contribution from the background is quite small in the vicinity of E_F, the estimated gap size from the fitting should not be affected by the background.

Supplementary Note 8. Accuracy of the calibrated Fermi level positions

Since the existence of superconducting gap nodes has been discussed in the present study, it is extremely important to accurately determine the Fermi level ($E_{\rm F}$) positions. As described in Methods section, we measured the Fermi edge of a gold film evaporated close to the sample holder and fitted the energy distribution curve (EDC) at each detector channel to a Gaussian-convoluted Fermi-Dirac function. Then, the obtained $E_{\rm F}$ positions were used as reference to calibrate the $E_{\rm F}$ positions of the spectra of measured samples. In order to evaluate the accuracy of the $E_{\rm F}$ positions, we have measured a gold film evaporated on a sample holder and calibrated the $E_{\rm F}$ positions using the reference. The $E_{\rm F}$ position thus calibrated for each detector channel is shown in Supplementary Figure 7. The average value for all the channels is deduced to be ~ 110 μeV, corresponding to the systematic error between the reference and the gold film evaporated on the sample holder, and the standard deviation is ~ 120 μeV, corresponding to the statistical error. Based on these results, we concluded that we could determine the $E_{\rm F}$ positions with an accuracy of ~ 200 μeV, which was used as the error bar of the superconducting gap size. We have also evaluated the temporal stability of $E_{\rm F}$ positions during several hours, and deduced it to be $\sim \pm 100$ μeV [10].

Supplementary References

[1] Damascelli, A., Hussain, Z. & Shen, Z.-X. Angle-resolved photoemission studies of the cuprate superconductors. *Rev. Mod. Phys.* **75**, 473–541 (2003).

[2] Kim, Y. *et al.* Electronic structure of detwinned $BaFe_2As_2$ from photoemission and first principles. *Phys. Rev. B* **83**, 064509 (2011).

[3] Shimojima, T. *et al.* Lifting of xz/yz orbital degeneracy at the structural transition in detwinned FeSe. *Phys. Rev. B* **90**, 121111 (2014).

[4] Suzuki, Y. *et al.* Momentum-dependent sign inversion of orbital order in superconducting FeSe. *Phys. Rev. B* **92**, 205117 (2015).

[5] Tokunaga, M., Kihara, T., Mizuguchi, Y. & Takano, Y. Field-Induced Magnetostructural Transitions in Antiferromagnetic $Fe_{1+y}Te_{1-x}S_x$. *J. Phys. Soc. Jpn.* **81**, 063703 (2012).

[6] Tanatar, M. A. *et al.* Origin of the Resistivity Anisotropy in the Nematic Phase of FeSe. *Phys. Rev. Lett.* **117**, 127001 (2016).

[7] Watashige, T. *et al.* Evidence for time-reversal symmetry breaking of the superconducting state near twin-boundary interfaces in FeSe revealed by scanning tunneling spectroscopy. *Phys. Rev. X* **5**, 031022 (2015).

[8] Matsui, H. *et al.* BCS-Like Bogoliubov Quasiparticles in High-T_c Superconductors Observed by Angle-Resolved Photoemission Spectroscopy. *Phys. Rev. Lett.* **90**, 217002 (2003).

[9] Shimojima, T. *et al.* Orbital-Independent Superconducting Gaps in Iron Pnictides. *Science* **332**, 564–567 (2011).

[10] Ota, Y. *et al.* Unconventional Superconductivity in the BiS_2-Based Layered Superconductor $NdO_{0.71}F_{0.29}BiS_2$. *Phys. Rev. Lett.* **118**, 167002 (2017).
